# Prevalence of delirium among patients with advanced cancer: a systematic review and meta-analysis

**DOI:** 10.3389/fneur.2026.1784653

**Published:** 2026-05-13

**Authors:** Linshan Jiang, Zhongyin Zhang, Xiaojun Liu, Yanxing Jiang, Qianqian Mou

**Affiliations:** 1 Department of Pharmacy, West China Hospital, Sichuan University, Chengdu, Sichuan, China; 2 Department of Rehabilitation Medicine, West China Hospital, Sichuan University, Chengdu, Sichuan, China; 3 Clinical Trial Center, West China Hospital, West China School of Nursing, Sichuan University, Chengdu, Sichuan, China

**Keywords:** advanced cancer, delirium, epidemiology, meta-analysis, prevalence

## Abstract

**Background:**

Delirium is a prevalent complication experienced by patients with advanced cancer and is associated with various adverse outcomes. Although delirium is widely reported among patients with advanced cancer, its estimated prevalence shows considerable heterogeneity across studies. This study aimed to determine the prevalence of delirium among patients with advanced cancer.

**Methods:**

A comprehensive literature search was conducted across ten major electronic databases from their inception to September 15, 2025. Data extraction was undertaken using a structured data collection form, and methodological quality was assessed using the Joanna Briggs Institute (JBI) critical appraisal tool for prevalence studies. A random-effects model was used to estimate the pooled prevalence of delirium. Heterogeneity was evaluated using the *I*^2^ statistic, with subgroup analyses performed to explore its potential sources.

**Results:**

A total of 17 studies comprising 9,007 patients with advanced cancer yielded a pooled delirium prevalence of 35.6% (95% CI: 27.2–44.1). 13 studies were appraised as having a low overall risk of bias, while 4 were considered to have a moderate risk of bias. The results of subgroup analysis indicated that the pooled prevalence of delirium among patients with advanced cancer exhibited significant variation depending on the assessment tool employed.

**Conclusion:**

Our findings indicated that delirium is highly prevalent among patients with advanced cancer, highlighting the necessity for early screening, prevention, and treatment. However, these results should be interpreted with caution due to limitations such as the small sample size and considerable heterogeneity. Moreover, the inclusion of only three studies from developing countries limits generalizability to low-resource settings.

**Systematic review registration:**

https://www.crd.york.ac.uk/PROSPERO/view/CRD420251167526.

## 1 Introduction

Cancer remains a primary public health challenge worldwide (1). Many patients with cancer are diagnosed at an advanced stage due to non-specific early symptoms and the lack of generalized screening (2), at which point the disease is usually irreversible and survival is predictable (3). At this point, the goal of treatment was redefined as the effective control of symptoms and the relief of suffering, with the overarching aim of preserving the patient’s quality of life as fully as possible (4). However, the pursuit of this objective is frequently complicated by the occurrence of delirium, a common and distressing psychiatric complication experienced by patients with advanced cancer (5).

Delirium is a complex syndrome characterized by an acute onset and encompassing disturbances in attention, orientation, and consciousness (6). It is one of the common symptoms experienced by patients with advanced cancer (7). Not only does this acute confusion intensify patient suffering, but it also poses significant challenges for clinical symptom management and palliative care (8). Furthermore, available evidence indicates that delirium is intimately associated with multiple adverse outcomes in patients with advanced cancer, including worsening functional status, prolonged hospital stays, higher financial burden and mortality (9, 10). More critically, delirium is substantially preventable and generally reversible, affording significant opportunities to optimize patient outcomes (11). However, delirium among patients with advanced cancer remains frequently under-diagnosed, mainly due to its nonspecific symptoms, inadequate clinical screening, and attribution bias regarding disease progression (12). These elements converge to highlight the urgent need for enhanced systematic screening and early intervention for delirium within this population.

The accurately estimated prevalence of delirium among patients with advanced cancer serves as crucial evidence for clinicians to establish effective prevention and treatment strategies. Although the clinical relevance of delirium in patients with advanced cancer has been universally acknowledged, existing studies have reported considerable variation in its specific prevalence, with findings ranging from 7 to 70% [(9, 13); Lawlor et al., 2020]. Potential reasons contribute to this significant heterogeneity, including the variable characteristics of the cohorts, the different instruments used to assess delirium, and the diverse study designs. A comprehensive systematic synthesis of these prevalence data is lacking, which makes it challenging to derive an accurate and representative pooled estimate. To address this gap, we conducted this systematic review and meta-analysis to answer the following question:

1. What is the pooled prevalence of delirium among patients with advanced cancer?
2. Is the pooled prevalence of delirium among patients with advanced cancer affected by economic status, settings, assessment tools, or diagnostic criteria?

## 2 Materials and methods

This study was conducted in accordance with the Preferred Reporting Items for Systematic Reviews and Meta-Analyses (PRISMA) guidelines (14). The study protocol was registered in PROSPERO (CRD420251167526).

### 2.1 Search strategy

A comprehensive literature search was conducted in ten electronic databases (PubMed, Web of Science, Scopus, EMBASE, CINAHL, Cochrane Library, CNKI, WanFang, VIP, and CBM) from inception until September 15, 2025. The initial terms included “neoplasms,” “cancer,” “advanced cancer,” “delirium,” and “prevalence.” The search strategies utilized Boolean operators to link MeSH terms with corresponding synonyms. Furthermore, the reference lists of included articles were screened to identify additional relevant publications. The search strategies for all databases are available in the Supplementary Table S1.

### 2.2 Study selection

The inclusion criteria were as follows: (1) participants were adult patients (≥18 years) with advanced cancer; (2) a cross-sectional or cohort study design was employed; (3) delirium was determined using validated tools or diagnostic criteria; (4) a calculable prevalence of delirium was reported. These studies were excluded if they were: (1) reviews, case reports, comments, editorials, or conference abstracts; (2) not published in English or Chinese; (3) unavailable in full text or contained overlapping data. If multiple publications were identified as originating from the same underlying data-set, only the one with the largest sample size was retained for analysis.

Two researchers (LSJ and ZYZ) independently screened the titles and abstracts of the initial literature. After the removal of duplicates, the full texts of the remaining records were assessed for eligibility against the predetermined inclusion criteria. The inter-rater reliability was assessed using kappa coefficient, yielding values of 0.86 for title and abstract screening and 0.91 for full-text review, indicating excellent agreement.

### 2.3 Data extraction

Data extraction was performed using a structured Excel form to maintain consistency. Two reviewers (LSJ and ZYZ) independently extracted multiple data from each included study, including first author, publication year, country, study design, setting, sample size, mean age, proportion of females, assessment tool, diagnostic criteria, and prevalence of delirium. A cross-checking procedure was conducted after data extraction to verify accuracy and resolve discrepancies.

### 2.4 Quality assessment

The risk of bias for each study was independently assessed by two reviewers using the Joanna Briggs Institute (JBI) critical appraisal tool for prevalence studies (15). The tool consists of nine items, each requiring a determination of “yes,” “no,” or “unclear/not applicable.” Studies were ultimately classified into three categories based on the percentage of entries rated “yes”: low risk (≥70%), moderate risk (50–69%), or high risk (≤49%) (1). Any disagreements that arose during the review process were directed to a third reviewer. A final consensus was then achieved through collective discussion.

### 2.5 Statistical analysis

Statistical analyses were conducted using Stata 15.0. A random-effects model was employed to determine the pooled prevalence of delirium in patients with advanced cancer and their corresponding 95% confidence intervals (CIs). In addition, we calculated the 95% prediction interval (PI) to estimate the range within which the true prevalence of a future individual study would fall. The random-effects model assumes that the true effect varies across studies and accounts for both within-study and between-study variance (16). This approach is considered more appropriate when substantial heterogeneity is expected, as it provides a more conservative estimate with wider confidence intervals and allows generalization beyond the included studies. The heterogeneity among the included studies was assessed by the *I*^2^ statistic. An *I*^2^ value ≥50% was considered to demonstrate substantial heterogeneity (17). The subgroup analyses based on economic status, geographic region, study design, setting, assessment tool, diagnostic criteria, and risk of bias were employed to identify the potential sources of heterogeneity. Furthermore, univariate meta-regression analyses were conducted to assess the association between covariates (sample size, publication year, economic status, geographic region, study design, setting, assessment tool, diagnostic criteria, and risk of bias) and the estimated prevalence. Publication bias was evaluated through a visual funnel plot and Egger’s test (18). Sensitivity analysis with the leave-one-out method was used to examine the robustness of the results (19). In this study, *p* < 0.05 was considered statistically significant (two-sided).

## 3 Results

### 3.1 Literature search and selection results

**Figure 1:**
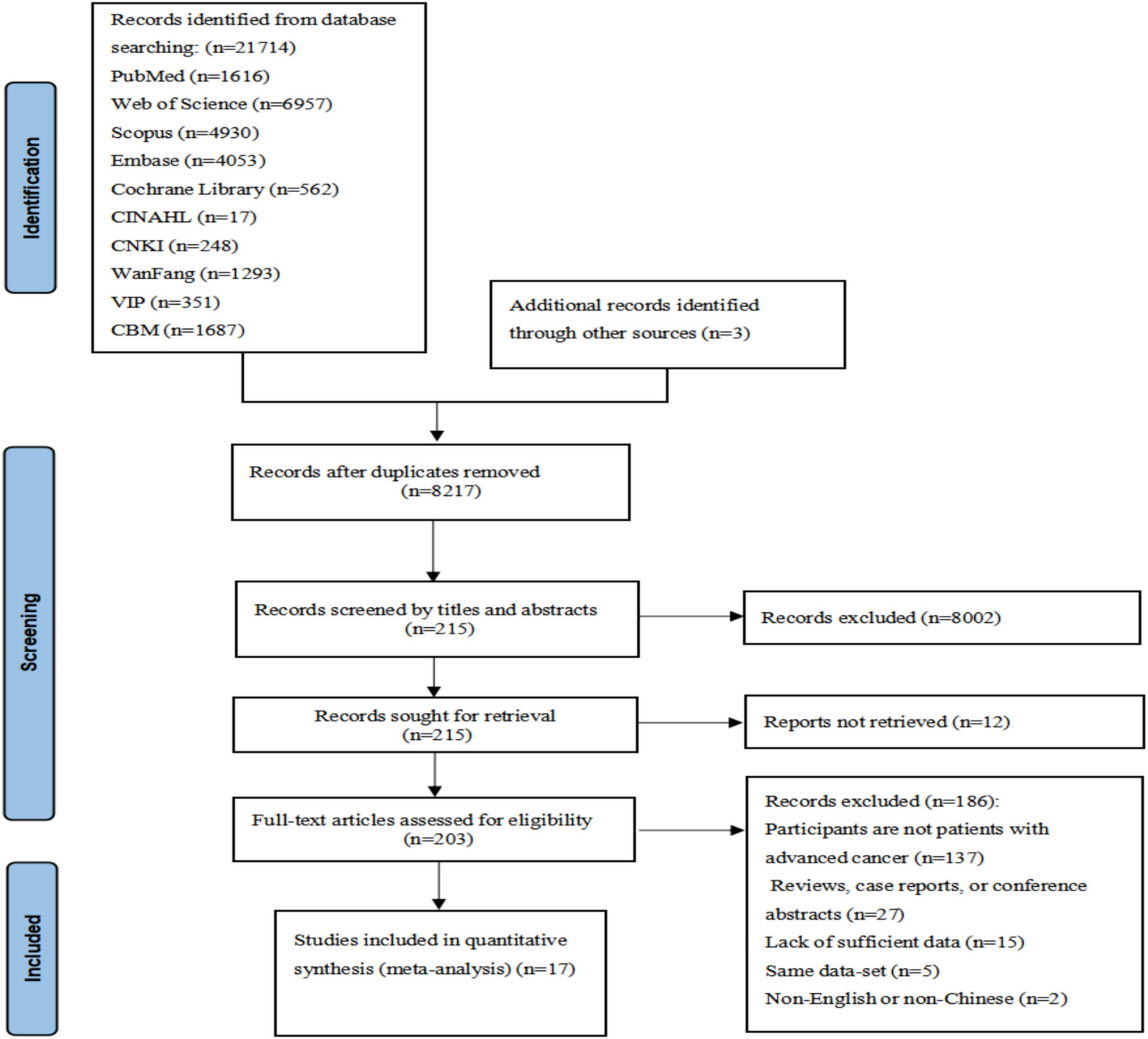
Flow diagram of study selection.

The initial search yielded 21,712 publications, with three additional records identified from reference lists. After eliminating duplication, 8,217 records were screened by title and abstract, of which 203 underwent full-text review. Finally, 17 studies met all inclusion criteria and were included. The screening process is detailed in Figure 1.

### 3.2 Characteristics of included studies

The 17 included studies [(8, 9, 11–13, 20–31)] encompassed 9,007 patients with advanced cancer, published between 2000 and 2025. Additionally, these studies were conducted in Italy (*n* = 4), USA (*n* = 4), China (*n* = 2), Japan (*n* = 2), Korea (*n* = 2), Canada (*n* = 1), Spain (*n* = 1), and India (*n* = 1). Regarding study design, a total of 14 articles were cohort, 2 were cross-sectional. In terms of clinical setting, most studies (*n* = 13) enrolled their participants from acute palliative care units. The most commonly utilized assessment tool and diagnostic criteria were MDAS (*n* = 11) and DSM-4 (*n* = 5), respectively. However, the majority of studies (*n* = 7) did not reported explicit clinical diagnostic criteria for delirium. Detailed characteristics of the included studies are summarized in Table 1.

**Table 1:** Characteristics of included studies.

Study	Country	Study design	Setting	Sample size	Mean age (years)	Female (%)	Assessment tool	Diagnostic criteria	Prevalence (%)
Caraceni et al. (20)	Italy	Cohort	Acute palliative care unit	393	NR	47.83	CAM	DSM-3	27.7
Chishi et al. (11)	India	Cohort	Hospice ward	147	NR	38.78	MDAS	DSM-4	31.29
de la Cruz et al. (21)	USA	Cross-sectional	Acute palliative care unit	556	56.51 ± 13.85	51.44	MDAS	DSM-4	58.09
Elsayem et al. (9)	USA	Cross-sectional	Emergency department	243	NR	49.38	CAM and MDAS	NR	9.05
Guo et al. (5)	China	Cohort	Acute palliative care unit	592	61.80 ± 14.20	47.5	3D-CAM	DSM-5	31.76
Hamano et al. (8)	Japan	Cohort	Acute palliative care unit and palliative home care unit	2,829	72.40 ± 12.20	47.26	MDAS	DSM-5	23.75
Hui et al. (24)	USA	Cohort	Acute palliative care unit	151	NR	62.91	MDAS	DSM-4	60.26
Hui et al. (23)	USA	Cohort	Acute palliative care unit	352	57	54.83	MDAS	NR	42.61
Kang et al. (25)	Korea	Cohort	Acute palliative care unit	102	NR	39.22	CAM	DSM-4	23.52
Kim et al. (13)	Korea	Cohort	Acute palliative care unit	2,314	66.30 ± 12.70	52.7	Physician and nurse diagnosis	DSM-5	7.13
Lawlor et al. (26)	Canada	Cohort	Acute palliative care unit	104	NR	39.42	MDAS	DSM-4	74.03
Llisterri-Sánchez et al. (27)	Spain	Cohort	Oncology unit	105	71.66 ± 4.10	44.76	CAM	DSM-5	63.81
Matsuo et al. (28)	Japan	Cohort	Acute palliative care unit	207	NR	52.66	CAM and MDAS	NR	16.91
Mercadante et al. (12)	Italy	Cohort	Acute palliative care unit	292	NR	42.12	MDAS	NR	8.21
Mercadante et al. (29)	Italy	Cohort	Acute palliative care unit	263	NR	NR	MDAS	NR	33.84
Pallotti et al. (30)	Italy	Cohort	Hospice ward and acute palliative care unit	227	NR	NR	MDAS	NR	38.77
Yang et al. (31)	China	Cross-sectional	Oncology unit	130	NR	45.38	CAM	NR	60.00

CAM, Confusion Assessment Method; 3D-CAM, 3-Minute Diagnostic Interview for Confusion Assessment Method; MDAS, memorial delirium assessment scale; DSM, Diagnostic and Statistical Manual of Mental Disorders.

### 3.3 Quality assessment results

Among the nine JBI appraisal items (Table 2), 10 studies (58.8%) had an appropriate sampling frame (Q1), 1 (5.9%) used adequate sampling methods (Q2), only 3 (17.6%) had adequate sample size (Q3), 16 (94.1%) described the study subjects and setting in detail (Q4), 16 (94.1%) had data analysis covering the identified sample sufficiently (Q5), all 17 studies (100%) used validated delirium assessment tools or explicit diagnostic criteria (Q6), measured delirium in a standard and reliable manner (Q7), and used appropriate statistical analysis (Q8), while 10 (58.8%) had adequate response rates or managed low response rates appropriately (Q9). Based on pre-specified percentage thresholds, 13 studies (76.5%) were rated as having an overall low risk of bias, 4 (23.5%) as moderate risk, and none as high risk. Inter-reviewer disagreement occurred in less than 5% of assessments (2 out of 34 items), primarily involving Q2 and Q9, and was resolved through discussion with a third reviewer.

**Table 2:** Summary of key quality issues across included studies.

Quality item	Number of studies rated “Yes” (%)
Appropriate sampling frame (Q1)	10 (58.8%)
Adequate sampling method (Q2)	1 (5.9%)
Adequate sample size (Q3)	3 (17.6%)
Detailed description of subjects and setting (Q4)	16 (94.1%)
Data analysis covering identified sample (Q5)	16 (94.1%)
Validated assessment tool or diagnostic criteria (Q6)	17 (100%)
Standard and reliable measurement (Q7)	17 (100%)
Appropriate statistical analysis (Q8)	17 (100%)
Adequate response rate or appropriately managed (Q9)	10 (58.8%)
Overall low risk of bias (≥70% “Yes”)	13 (76.5%)
Overall moderate risk of bias (50–69% “Yes”)	4 (23.5%)
Overall high risk of bias (≤49% “Yes”)	0 (0%)

### 3.4 Pooled prevalence of delirium in patients with advanced cancer

**Figure 2:**
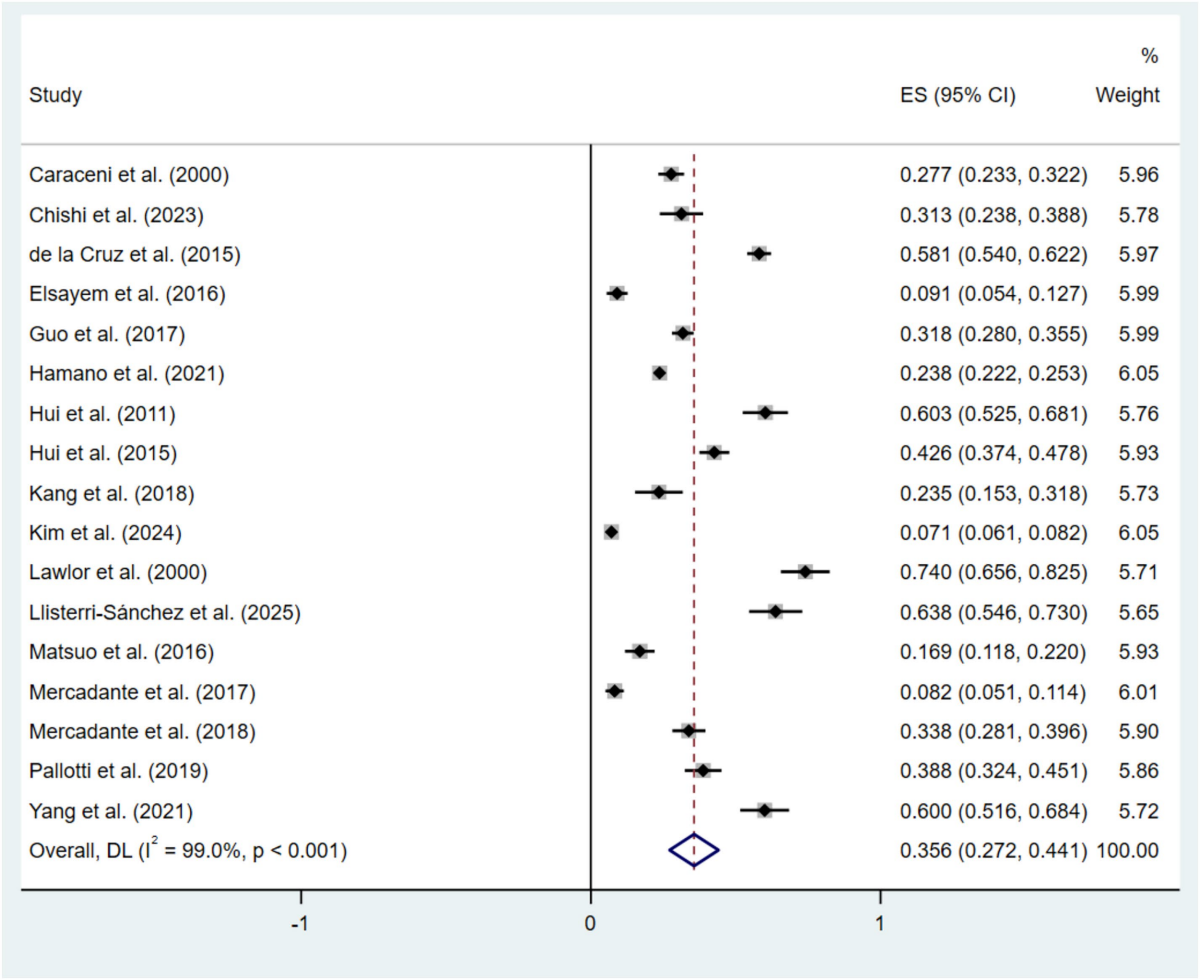
The pooled prevalence of delirium among patients with advanced cancer. Weights are from random-effects model.

In this study, 17 studies provided the prevalence of delirium among patients with advanced cancer, ranging from 7.13 to 74.03%. The random-effects model revealed that the pooled prevalence of delirium in patients with advanced cancer was 35.6% (95% CI: 27.2–44.1, *I*^2^ = 99.0%, *p* < 0.001) (Figure 2). The 95% prediction interval (PI) ranged from 0.0 to 72.8%, indicating that the true prevalence in a future individual study is expected to fall within this wide range, reflecting the substantial heterogeneity across settings.

### 3.5 Subgroup analysis and meta-regression

In the subgroup analysis based on assessment tools, the pooled prevalence of delirium screening using the CAM was significantly higher than other scales (*p* < 0.001). In contrast, stratified analysis by economic status, geographic region, study design, setting, diagnostic criteria, and risk of bias demonstrated no statistically significant differences in the pooled prevalence of delirium between the subgroups (*p* > 0.05). Detailed results of the subgroup analysis are depicted in Table 3, Supplementary Figures S1–S7.

**Table 3:** Subgroup analyses of the prevalence of delirium among patients with advanced cancer.

Subgroups	Number of included studies	Prevalence (%)	95% CI	*p* value
Economic status				0.511
Developed country	14	34.5	25.3–43.8	
Developing country	3	40.7	24.7–56.8	
Geographic region				0.267
Asia	7	27.4	17.3–37.4	
Europe	5	34.1	17.8–50.5	
North America	5	48.7	24.2–73.2	
Study design				0.674
Cross-sectional	3	42.3	5.4–79.2	
Cohort	14	34.2	25.8–42.5	
Setting				0.659
Inpatient palliative care unit	13	34.1	24.7–43.6	
Other units	6	40.9	12.5–69.2	
Assessment tool				<0.001
CAM	4	43.6	23.9–63.3	
MDAS	9	41.0	28.6–53.4	
CAM and MDAS	2	12.8	5.1–20.5	
3D-CAM	1	31.8	28.0–35.5	
Physician and nurse diagnosis	1	7.1	6.1–8.2	
Diagnostic criteria				0.257
DSM-based	10	39.9	28.0–51.7	
None	7	29.7	16.6–42.7	
Risk of bias				0.780
Low	13	36.5	26.7–46.3	
Moderate	4	33.0	10.3–55.7	

CAM, Confusion Assessment Method; 3D-CAM, 3-Minute Diagnostic Interview for Confusion Assessment Method; MDAS, memorial delirium assessment scale; CI, confidence interval; DSM, Diagnostic and Statistical Manual of Mental Disorders.

Univariable meta-regression analyses using logit-transformed proportions revealed no significant predictors of delirium prevalence (Table 4). Publication year, sample size, study design, economic status, geographic region, setting, assessment tool, diagnostic criteria, and risk of bias were not significantly associated with the prevalence (all *p* > 0.05).

**Table 4:** Meta-regression results.

Variables	Number of studies	Coefficient	Standard error	95%CI	*t* values	*p* values
Publication year	17	−0.419	0.0379	(−0.1226, 0.0389)	−1.11	0.287
Sample size	17	−0.5104	0.2486	(−1.0402, 0.0195)	−2.05	0.058
Study design (Ref: Cross-sectional)						
Cohort	14	−0.2653	0.7060	(−1.7700, 1.2394)	−0.38	0.712
Economic status (Ref: Developed country)						
Developing country	3	0.4186	0.6996	(−1.0725, 1.9098)	0.60	0.559
Geographic region (Ref: Asia)						
Europe	5	−0.9388	0.6263	(−2.2820, 0.4040)	−1.50	0.159
North America	5	−0.6264	0.6768	(−2.0779, 0.8251)	0.93	0.370
Setting (Ref: Inpatient palliative care unit)						
Other units	6	0.2651	0.6353	(−1.0891, 1.6193)	0.42	0.682
Assessment tool (Ref: CAM)						
MDAS	9	0.4736	1.0482	(−0.3248, −0.944)	0.45	0.659
CAM and MDAS	2	−1.1816	1.1522	(−3.6921, 1.3290)	−1.03	0.325
3D-CAM	1	0.3205	0.9861	(−1.8279, 2.4690)	0.33	0.751
Physician and nurse diagnosis	1	−1.8018	1.3213	(−4.6806, 1.0769)	−1.36	0.198
Diagnostic criteria (Ref: DSM-based)						
None	7	−0.5251	0.5320	(−1.6590, 0.6088)	−0.99	0.339
Risk of bias (Ref: Low)						
Moderate	4	−0.2343	0.6358	(−1.5894, 1.1209)	−0.37	0.718

### 3.6 Publication bias and sensitivity analysis

**Figure 3:**
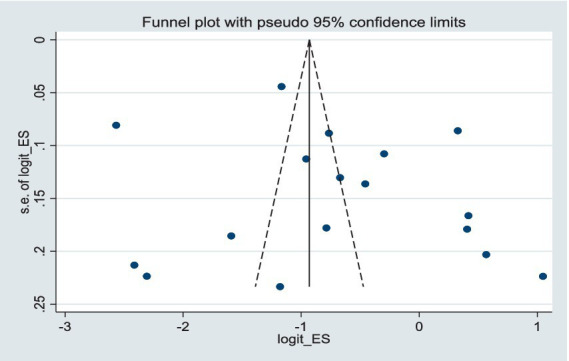
Funnel plot.

No significant publication bias was detected in the pooled prevalence of delirium, as assessed by funnel plot (Figure 3) and Egger’s regression test (*t* = 1.03, *p* = 0.318; Supplementary Figure S8). Furthermore, the leave-one-out sensitivity analysis confirmed that no single study disproportionately influenced the results, demonstrating the stability of the pooled prevalence (Supplementary Table S3). Three additional sensitivity analyses were conducted to assess the robustness of the pooled estimate. First, excluding studies conducted in developing countries (Supplementary Figure S9) yielded a pooled prevalence of 34.5% (95% CI: 25.3–43.8%; *I*^2^ = 99.1%), which was comparable to the main estimate of 35.6% (95% CI: 27.2–44.1%; *I*^2^ = 99.0%). Second, excluding studies that did not use validated assessment tools (Supplementary Figure S10) produced a pooled prevalence of 37.9% (95% CI: 28.8–46.9%; *I*^2^ = 98.4%). Third, excluding studies without clear diagnostic criteria for delirium (Supplementary Figure S11) gave a pooled prevalence of 39.9% (95% CI: 28.0–51.7%; *I*^2^ = 99.3%). All three sensitivity analyses yielded estimates similar to the main analysis, indicating that the overall prevalence finding is robust to the exclusion of studies from developing countries, those with unvalidated tools, or those with unclear diagnostic criteria.

## 4 Discussion

Delirium is intimately associated with mortality in patients with advanced cancer (26). However, existing evidence shows inconsistent estimates of the prevalence of delirium among patients with advanced cancer. This meta-analysis, synthesizing data from 9,007 participants across 8 countries, estimated a pooled prevalence of delirium in patients with advanced cancer at 35.6% (95% CI: 27.2–44.1%). Despite the extreme heterogeneity observed, the pooled estimate of 35.6% remains clinically meaningful, as all included studies reported prevalence substantially higher than general hospitalized populations and the lower bound of the confidence interval exceeds clinically relevant thresholds. However, this estimate should be interpreted as a central tendency across diverse contexts rather than a universal value, and clinicians should consider local prevalence and individual risk factors when applying these findings to practice. Furthermore, the finding highlights delirium not as a peripheral issue, but as a core component of the patients with advanced cancer. The high prevalence of delirium in patients with advanced cancer generally derives from the convergence of multiple physiological and psychological factors. Current research evidence suggests that terminal cancer can directly impair the central nervous system through brain metastases, parasitic syndromes, and systemic inflammation (32). Moreover, managing cancer-related pain in patients with advanced cancer often requires centrally acting medications, such as opioid analgesics and benzodiazepines (33). These agents readily cross the blood–brain barrier and have well-documented adverse effects that could contribute to cerebral dysfunction and delirium (34). Moreover, it is noteworthy that sleep disorders are prevalent among patients with advanced cancer (35). Not only do they generate emotional distress and cognitive impairment, but their long-term implications also disrupt neurotransmitter balance, thereby serving as a significant vulnerability for delirium onset (36). Therefore, our findings support routine delirium screening using the CAM tool for all patients with advanced cancer admitted to inpatient palliative care or acute oncology wards, with confirmatory DSM-based assessment for screen-positive cases. In outpatient or lower-risk settings, targeted screening for high-risk patients is recommended.

The stratified analysis based on economic status revealed no significant difference in the pooled prevalence of delirium among patients with advanced cancer between developed and developing countries. This negative finding may be attributed to the insufficient statistical power of the current study. Given that only three studies conducted in developing countries were included in the analysis, the available data may be inadequate to detect any actual differences between groups. Moreover, the wide confidence interval for developing countries overlaps substantially with the developed country estimate, precluding any conclusion of equivalence. Consequently, the findings of this meta-analysis primarily reflect advanced cancer populations in developed countries with established palliative care infrastructure. Generalizability to low-resource settings remains uncertain and requires further investigation. It is also important to recognize that healthcare infrastructure, delirium screening practices, and access to palliative care vary substantially across countries. In resource-constrained settings, underdiagnosis of delirium may be more prevalent due to insufficient training of healthcare professionals, lack of standardized screening tools, and limited access to specialist consultation. Consequently, the true prevalence of delirium in these settings may be either overestimated or underestimated depending on the interplay of these contextual factors. Therefore, the findings of this meta-analysis may not be generalizable to low-resource settings, where healthcare infrastructure, screening practices, and palliative care access differ markedly from the predominantly high-income country contexts represented in this study.

Palliative care plays an indispensable role in the administration of end-stage cancer care (37). It aims to reduce suffering through the early identification and management of pain, along with other physical and psychological concerns, thereby improving the quality of life for patients (38). Our findings demonstrated that the pooled prevalence of delirium among patients with advanced cancer receiving care in inpatient palliative care units was not significantly different from that observed in other clinical settings. These findings suggest that delirium prevalence is determined not by the type of clinical ward, but by the underlying acuity of a patient’s illness. For patients with advanced cancer, the magnitude of physiological disturbance converges toward a threshold that is sufficiently high to induce delirium, whether they are located in an oncology ward, emergency department, or APCU, thus explaining the similar rates observed across these settings. Therefore, healthcare professionals must be aware that the prevention and management of delirium should not be confined to specialized palliative care units but be integrated into all clinical settings providing care for patients with advanced cancer.

There are a variety of tools available for assessing delirium in patients with advanced cancer, yet their diagnostic efficacy remains varied. Our subgroup analysis showed that the pooled prevalence derived using the CAM was significantly higher than that based on physician and nurse diagnosis. This discrepancy reflects differences in detection capability, not necessarily true prevalence. CAM was designed as a screening tool with high sensitivity (39), but higher sensitivity does not equal higher true prevalence. It may capture borderline or subsyndromal delirium (40). Moreover, the distribution of assessment tools across studies was not random. CAM-based studies may have been conducted in higher-risk settings, introducing confounding. Without a gold standard reference test applied across all studies, we cannot determine which tool is most accurate. Therefore, we propose interpreting the CAM-based estimate (43.6%) as an upper bound (maximizing sensitivity), while estimates from MDAS or DSM-based interviews provide a more conservative lower bound (prioritizing specificity). To address this, we conducted a sensitivity analysis excluding studies using unvalidated tools; the pooled prevalence using only validated instruments was 37.9% (95% CI: 28.8–46.9%). Clinically, regardless of which estimate is used, all validated tools confirm that delirium is a common complication in advanced cancer, and reliance on unstructured clinical observation substantially underestimates its burden. Therefore, we recommend standardized screening with a high-sensitivity instrument, while recognizing that screening-positive cases require confirmatory diagnostic assessment to avoid overdiagnosis.

The DSM-5 is regarded as one of the gold standards for diagnosing delirium (41). In contrast to screening tools, DSM-based diagnostic interviews prioritize specificity over sensitivity, requiring the presence of all core diagnostic features along with clinically significant impairment. This approach minimizes false positives but may result in underdiagnosis by excluding milder or atypical presentations. The subgroup analysis in this study suggested that no statistically significant differences were detected in the pooled prevalence of delirium among patients with advanced cancer diagnosed using different versions of DSM criteria. This phenomenon could be explained by the consistency of the core diagnostic elements of delirium across the various versions of the DSM. The fundamental components of delirium diagnosis have remained unchanged, including acute impairment of consciousness, cognitive changes, and a clearly identifiable physical cause (42). As a result, despite minor terminological adjustments, different editions of the DSM exhibit a high degree of consistency in identifying primary cases of delirium. Consequently, prevalence estimates based on alternative versions show no significant divergence. Notably, our findings confirmed the long-term stability of the diagnostic framework based on the core criteria of the DSM, laying a theoretical foundation for achieving diagnostic consistency across institutions and physician groups. Therefore, it is recommended that clinical training focusing on this core criteria framework should be implemented to improve healthcare professionals’ ability to identify and diagnose delirium.

### 4.1 Strengths and limitations

Given that the precise prevalence of delirium in patients with advanced cancer remains an under-explored area, we conducted this meta-analysis to address the significant gap. This study has several prominent strengths. First of all, this is the first meta-analysis to quantitatively synthesize the prevalence of delirium in patients with advanced cancer. In addition, an extensive search strategy was implemented, with multiple electronic databases searched. Rigorous quality control measures were employed throughout study selection, data extraction, and quality assessment, ensuring the scientific rigor and reliability of the findings.

Several limitations should be considered in this study. First, as with other prevalence meta-analyses, significant heterogeneity was observed among the included studies. However, no conclusive evidence exists to explain the potential causes, so the findings should be given careful consideration. Second, a considerable proportion of the included studies (7 out of 17) did not report clear diagnostic criteria for delirium, which may introduce ascertainment bias, thereby compromising the accuracy and comparability of prevalence estimates. Third, the majority of the included studies were conducted in developed countries, with only three studies from developing countries. This geographic skew means that our findings primarily reflect patients with advanced cancer in high-income settings with established palliative care infrastructure. Whether these results apply to low-resource regions, where healthcare resources, medication access, and delirium screening practices may differ substantially, remains unknown. Future studies from developing countries are urgently needed to address this evidence gap. Fourth, it is acknowledged that relevant unpublished or gray literature from developing countries may exist but was not captured by our search strategy, which was focused on peer-reviewed databases. Regional databases and non-English and non-Chinese sources should be incorporated in future meta-analyses to address this gap. Finally, the small sample size of patients with advanced cancer analyzed in this study may have contributed to potential bias in the results.

## 5 Conclusion

In conclusion, this meta-analysis demonstrated that delirium was prevalent among patients with advanced cancer, with a prevalence of 35.6%. These findings provide a foundation for designing targeted screening approaches, specifically routine CAM screening for hospitalized patients with advanced cancer and confirmatory DSM-based assessment for positives, as well as non-pharmacological intervention strategies focused on medication review, hydration, and family support. However, these results should be interpreted with caution due to limitations including the small sample size, considerable heterogeneity, and the fact that only three of the 17 included studies were from developing countries, which limits generalizability to low-resource settings.

## Data Availability

The original contributions presented in the study are included in the article/Supplementary material, further inquiries can be directed to the corresponding author.
