# Supplementary material for: Prevalence of delirium among patients with advanced cancer: a systematic review and meta-analysis
Source: Front Neurol. 2026 May 13;17:1784653. doi: 10.3389/fneur.2026.1784653 (PMC13214514; doi:10.3389/fneur.2026.1784653)
Supplement: Supplementary file 1 [file Table_1.DOCX]

**Supplemental Materials**

Table S1. The search strategies (Date Run: 1/10/2025).

| **Databases** | **Step** | **Search Strategies** | **Results** |
| --- | --- | --- | --- |
| PubMed | #1 | "[neoplasms](https://www.ncbi.nlm.nih.gov/mesh/68009369)"[Mesh Terms] Sort by: Most Recent | 4160178 |
|  | #2 | "cancer"[Title/Abstract] OR "tumor"[Title/Abstract] OR "tumour"[Title/Abstract] OR "carcinoma"[Title/Abstract] OR "malignancy"[Title/Abstract] OR "advanced cancer"[Title/Abstract] Sort by: Most Recent | 3917745 |
|  | #3 | "delirium"[Mesh Terms] Sort by: Most Recent | 14638 |
|  | #4 | "confusion"[Mesh Terms] Sort by: Most Recent | 19511 |
|  | #5 | "delirium"[Title/Abstract] OR "confusion"[Title/Abstract] OR "encephalopathy"[Title/Abstract] OR "acute confusion"[Title/Abstract]  Sort by: Most Recent | 128433 |
|  | #6 | "prevalence"[Mesh Terms] Sort by: Most Recent | 378782 |
|  | #7 | "incidence"[Mesh Terms] Sort by: Most Recent | 326101 |
|  | #8 | "e[pidemiology](https://www.ncbi.nlm.nih.gov/mesh/68004813)"[Mesh Terms] Sort by: Most Recent | 29394 |
|  | #9 | "prevalence"[Title/Abstract] OR "incidence"[Title/Abstract] OR "e[pidemiology](https://www.ncbi.nlm.nih.gov/mesh/68004813)"[Title/Abstract] Sort by: Most Recent | 2083159 |
|  | #10 | #1 OR #2 Sort by: Most Recent | 5413831 |
|  | #11 | #3 OR #4 OR #5 Sort by: Most Recent | 133045 |
|  | #12 | #6 OR #7 OR #8 Sort by: Most Recent | 2266969 |
|  | #13 | #10 AND #11 AND #12 Sort by: Most Recent | 1616 |
| Web of Science | #1 | **Ts=(**[neoplasms](https://www.ncbi.nlm.nih.gov/mesh/68009369)**) OR TS=(cancer**) OR TS=(tumor) OR Ts=(tumour) OR Ts=(carcinoma) OR Ts=(malignancy) OR Ts=(advanced cancer) | 9701970 |
|  | #2 | **Ts=(delirium) OR Ts=(confusion) OR** TS=(encephalopathy) OR **TS=(**acute confusion) | 335912 |
|  | #3 | TS=(prevalence) OR **TS=(incidence) OR TS=(**e[pidemiology](https://www.ncbi.nlm.nih.gov/mesh/68004813)) | 5602478 |
|  | #4 | #1 AND #2 AND #3 | 6957 |
| Scopus | #1 | TITLE-ABS-KEY ("[neoplasms](https://www.ncbi.nlm.nih.gov/mesh/68009369)" OR "cancer" OR "tumor" OR "tumour" OR "carcinoma" OR "malignancy" OR "advanced cancer") | 6844608 |
|  | #2 | TITLE-ABS-KEY ("delirium" OR "confusion" OR "encephalopathy" OR "acute confusion") | 309368 |
|  | #3 | TITLE-ABS-KEY ("incidence" OR "prevalence" OR "e[pidemiology](https://www.ncbi.nlm.nih.gov/mesh/68004813)") | 3685431 |
|  | #4 | #1 AND #2 AND #3 | 4930 |
| EMBASE | **#1** | **'**[neoplasms](https://www.ncbi.nlm.nih.gov/mesh/68009369)**'/exp** | 6906944 |
|  | **#2** | 'cancer':ti,ab,kw OR 'tumor':ti,ab,kw OR 'tumour':ti,ab,kw OR 'carcinoma':ti,ab,kw OR 'malignancy':ti,ab,kw OR 'advanced cancer':ti,ab,kw | 5588019 |
|  | #3 | 'delirium**':ti,ab,kw OR 'confusion':ti,ab,kw OR '**encephalopathy**':ti,ab,kw OR '**acute confusion**':ti,ab,kw** | 201673 |
|  | #4 | 'prevalence**':ti,ab,kw OR 'incidence':ti,ab,kw OR '**e[pidemiology](https://www.ncbi.nlm.nih.gov/mesh/68004813)**':ti,ab,kw** | 3031628 |
|  | #5 | #1 OR #2 | 7908059 |
|  | #6 | #3 AND #4 AND #5 | 4053 |
| Cochrane Library | #1 | Mesh descriptor: [Neoplasms] explode all trees | 129812 |
|  | #2 | (cancer):ti,ab,kw OR (tumor):ti,ab,kw OR (tumour):ti,ab,kw OR (carcinoma):ti,ab,kw OR (malignancy):ti,ab,kw OR (advanced cancer):ti,ab,kw | 270977 |
|  | #3 | Mesh descriptor: [Delirium] explode all trees | 1781 |
|  | #4 | (delirium)**:ti,ab,kw OR (confusion**)**:ti,ab,kw OR (**encephalopathy)**:ti,ab,kw OR (**acute confusion)**:ti,ab,kw** | 14602 |
|  | #5 | (prevalence)**:ti,ab,kw OR (incidence**)**:ti,ab,kw OR (**e[pidemiology](https://www.ncbi.nlm.nih.gov/mesh/68004813))**:ti,ab,kw** | 257886 |
|  | #6 | #1 OR #2 | 297259 |
|  | #7 | #3 OR #4 | 14602 |
|  | #8 | #5 AND #6 AND #7 | 562 |
| CINAHL | S1 | **TI** [neoplasms](https://www.ncbi.nlm.nih.gov/mesh/68009369) **OR TI cancer** OR TI tumor OR TI tumour OR TI carcinoma OR TI malignancy OR TI advanced cancer | 543110 |
|  | S2 | TI delirium **OR TI confusion OR TI** encephalopathy **OR TI** acute confusion | 16363 |
|  | S3 | TI prevalence **OR TI incidence OR TI** e[pidemiology](https://www.ncbi.nlm.nih.gov/mesh/68004813) | 123109 |
|  | S4 | S1 AND S2 AND S3 | 17 |
| CNKI |  | (SU="肿瘤"+ "晚期肿瘤" + "恶性肿瘤" + "癌症" + "晚期癌症") AND (SU="谵妄" + "脑功能障碍") | 248 |
| WanFang |  | 题名或关键词:(肿瘤 OR 晚期肿瘤 OR 恶性肿瘤 OR 癌症OR 晚期癌症) and 题名或关键词:(谵妄 OR 脑功能障碍) | 1293 |
| VIP |  | (题名或关键词=肿瘤 OR 晚期肿瘤 OR 恶性肿瘤OR 癌症 OR 晚期癌症) and (题名或关键词=谵妄 OR 脑功能障碍) | 351 |
| CBM |  | ("肿瘤"[常用字段:智能] OR "晚期肿瘤"[常用字段:智能] OR "恶性肿瘤"[常用字段:智能] OR "癌症"[常用字段:智能] OR "晚期癌症"[常用字段:智能]) AND( "谵妄"[常用字段:智能] OR "脑功能障碍"[常用字段:智能]) AND "发生率"[核心字段:智能]) | 1687 |

Table S2. Quality assessment results of included studies.

| Study | Q1 | Q2 | Q3 | Q4 | Q5 | Q6 | Q7 | Q8 | Q9 | Overall |
| --- | --- | --- | --- | --- | --- | --- | --- | --- | --- | --- |
| Caraceni et al. (2000) | Y | U | N | Y | Y | Y | Y | Y | Y | L |
| Chishi et al. (2023) | U | U | U | Y | Y | Y | Y | Y | Y | L |
| de la Cruz et al. (2015) | Y | U | U | Y | Y | Y | Y | Y | Y | L |
| Guo et al. (2025) | Y | U | Y | Y | Y | Y | Y | Y | Y | L |
| Elsayem et al. (2016) | Y | Y | Y | Y | Y | Y | Y | Y | N | L |
| Hamano et al. (2021) | Y | U | U | Y | Y | Y | Y | Y | Y | L |
| Hui et al. (2015) | Y | U | U | Y | Y | Y | Y | Y | N | L |
| Hui et al. (2015) | Y | U | U | Y | Y | Y | Y | Y | Y | L |
| Kang et al. (2018) | N | N | N | Y | Y | Y | Y | Y | Y | L |
| Kim et al. 2023 | Y | N | N | Y | Y | Y | Y | Y | Y | L |
| Lawlor et al. (2000) | N | N | N | Y | Y | Y | Y | Y | U | M |
| Llisterri-Sánchez et al. (2025) | N | N | N | Y | Y | Y | Y | Y | Y | L |
| Matsuo et al. (2016) | N | U | N | Y | U | Y | Y | Y | Y | M |
| Mercadante et al. (2017) | U | U | N | Y | Y | Y | Y | Y | U | M |
| Mercadante et al. (2018) | U | U | N | Y | Y | Y | Y | Y | U | M |
| Pallotti et al. (2019) | Y | N | Y | N | Y | Y | Y | Y | U | L |
| Yang et al. (2021) | Y | N | U | Y | Y | Y | Y | Y | U | L |

Notes: Y: Yes, N: No, U: Unclear. L: Low risk, M: Moderate risk, H: High risk.

Q1: Was the sample frame appropriate to address the target population?

Q2: Were study participants sampled in an appropriate way?

Q3: Was the sample size adequate?

Q4: Were the study subjects and the setting described in detail?

Q5: Was the data analysis conducted with sufficient coverage of the identified sample?

Q6: Were valid methods used for the identification of the condition?

Q7: Was the condition measured in a standard, reliable way for all participants?

Q8: Was there appropriate statistical analysis?

Q9: Was the response rate adequate, and if not, was the low response rate managed appropriately?

Each study was categorized by risk of bias as follows: high risk if ≤ 49% of items are responded to with "yes;" moderate risk if 50%-69% of items are responded to with "yes; "and low risk if ≥ 70% of items are responded to with "yes."


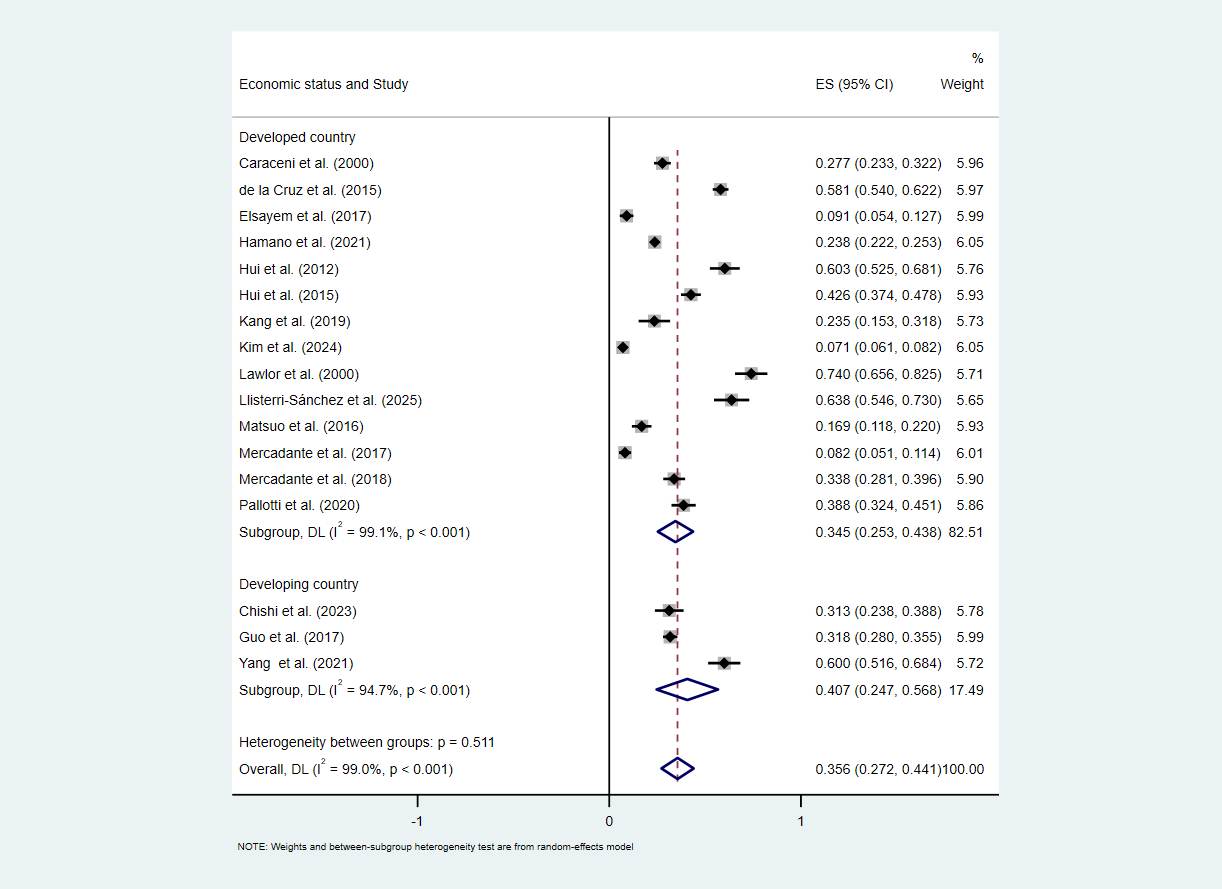


Figure S1. The pooled prevalence of delirium among patients with advanced cancer based on economic status.


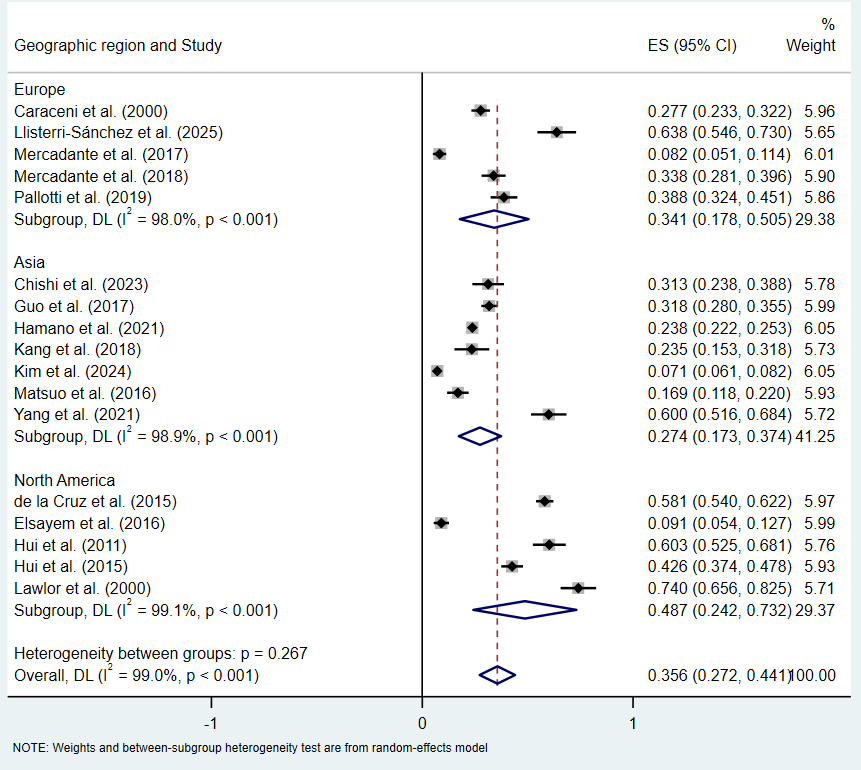


Figure S2. The pooled prevalence of delirium among patients with advanced cancer based on geographic region.


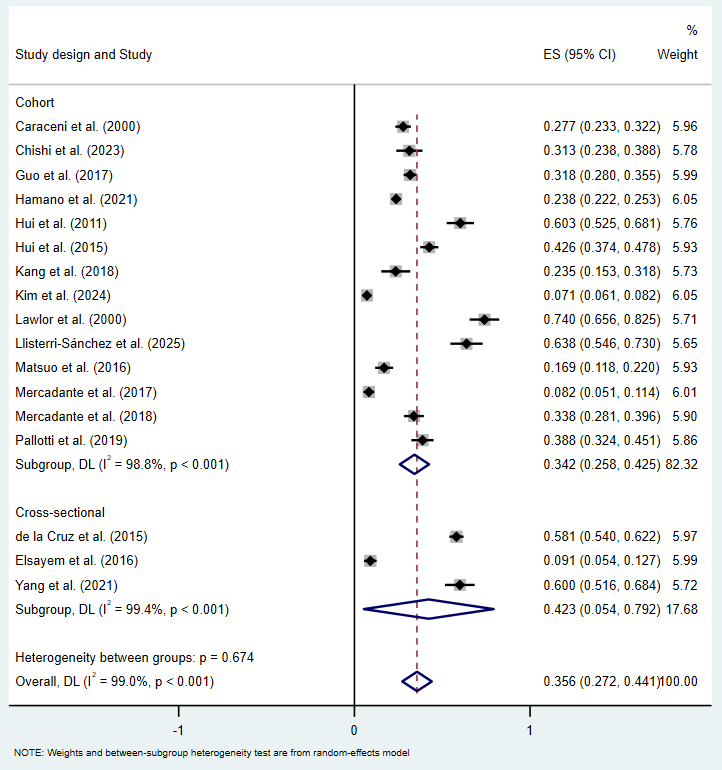


Figure S3. The pooled prevalence of delirium among patients with advanced cancer based on study design.


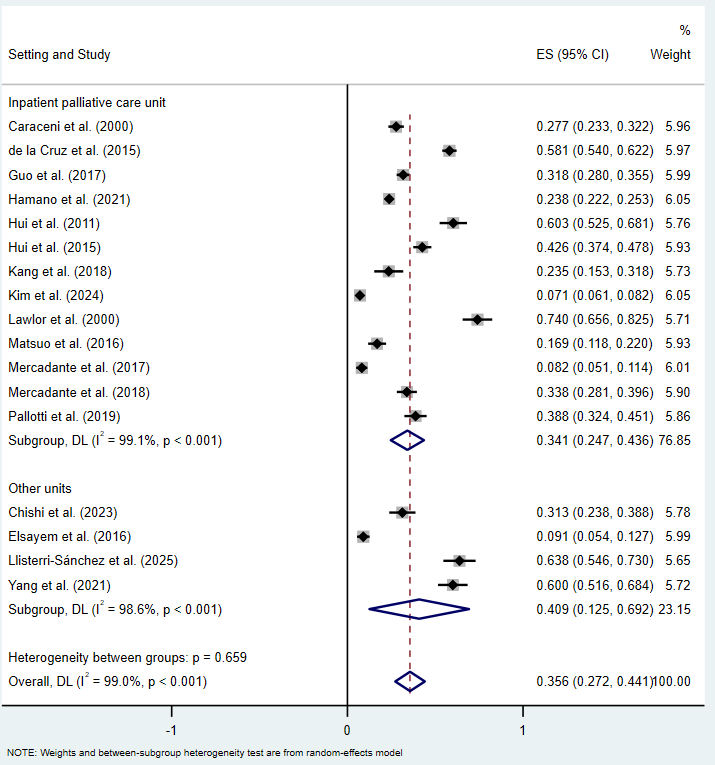


Figure S4. The pooled prevalence of delirium among patients with advanced cancer based on setting.


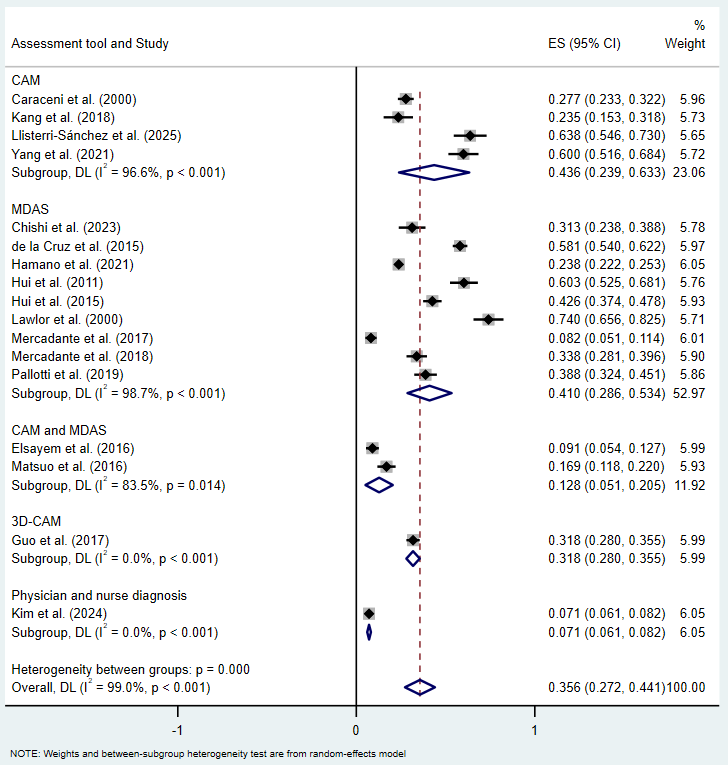


Figure S5. The pooled prevalence of delirium among patients with advanced cancer based on assessment tool.


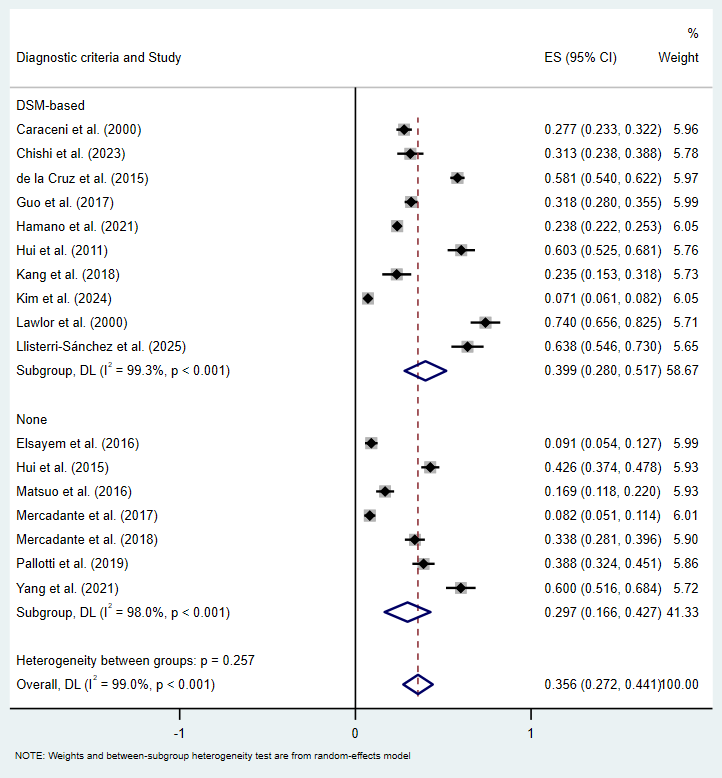


Figure S6. The pooled prevalence of delirium among patients with advanced cancer based on diagnostic criteria.


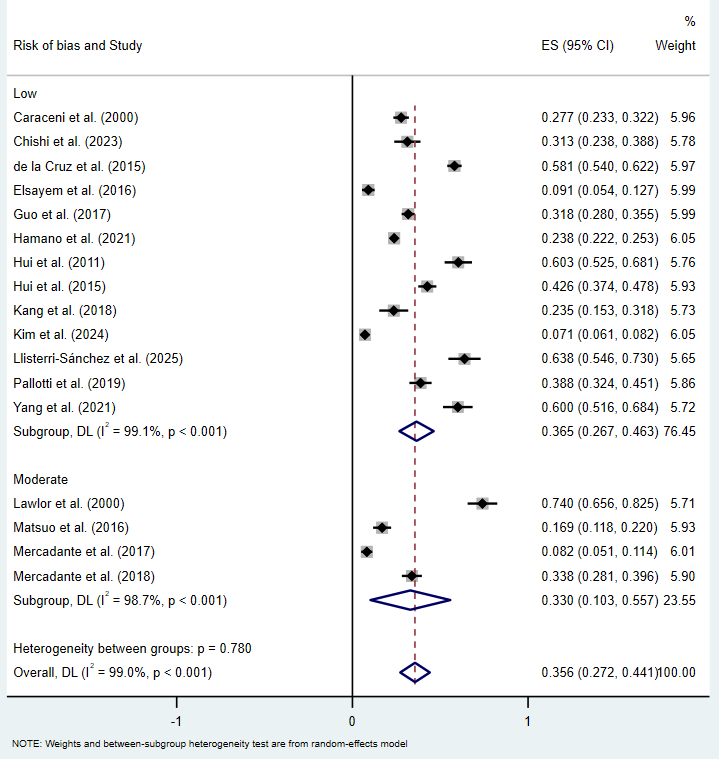


Figure S7. The pooled prevalence of delirium among patients with advanced cancer based on risk of bias.


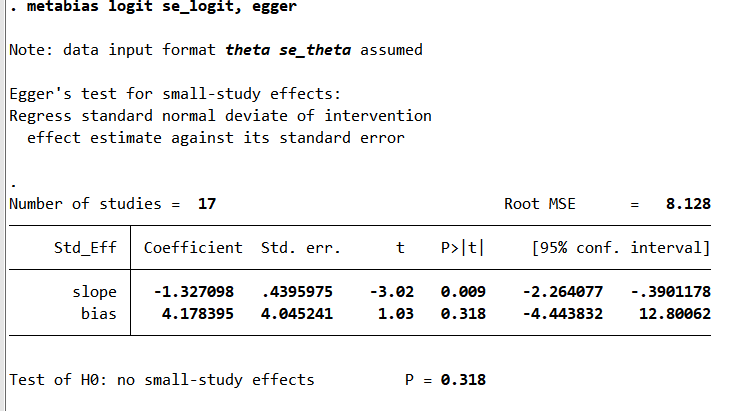


Figure S8. Egger's test results

Table S3. The results of the sensitivity analysis.

| **Study omitted** | **Prevalence (%)** | **95% CI** |
| --- | --- | --- |
| Caraceni et al. (2000) | 36.1% | 27.3% - 45.0% |
| Chishi et al. (2023) | 35.9% | 27.2% - 44.6% |
| de la Cruz et al. (2015) | 34.1% | 26.3% - 41.9% |
| Elsayem et al. (2016) | 37.3% | 28.4% - 46.3% |
| Guo et al. (2017) | 35.9% | 27.0% - 44.7% |
| Hamano et al. (2021) | 36.5% | 26.2% - 46.8% |
| Hui et al. (2011) | 34.1% | 25.7% - 42.5% |
| Hui et al. (2015) | 35.2% | 26.6% - 43.8% |
| Kang et al. (2018) | 36.4% | 27.6% - 45.1% |
| **Kim et al. (2024)** | **37.4%** | **29.1% - 45.8%** |
| Lawlor et al. (2000) | 33.3% | 25.0% - 41.5% |
| Llisterri-Sánchez et al. (2025) | 33.9% | 25.4% - 42.4% |
| Matsuo et al. (2016) | 36.8% | 28.0% - 45.7% |
| Mercadante et al. (2017) | 37.4% | 28.4% - 46.4% |
| Mercadante et al. (2018) | 35.7% | 27.0% - 44.5% |
| Pallotti et al. (2019) | 35.4% | 26.7% - 44.1% |
| Yang et al. (2021) | 34.1% | 25.6% - 42.6% |


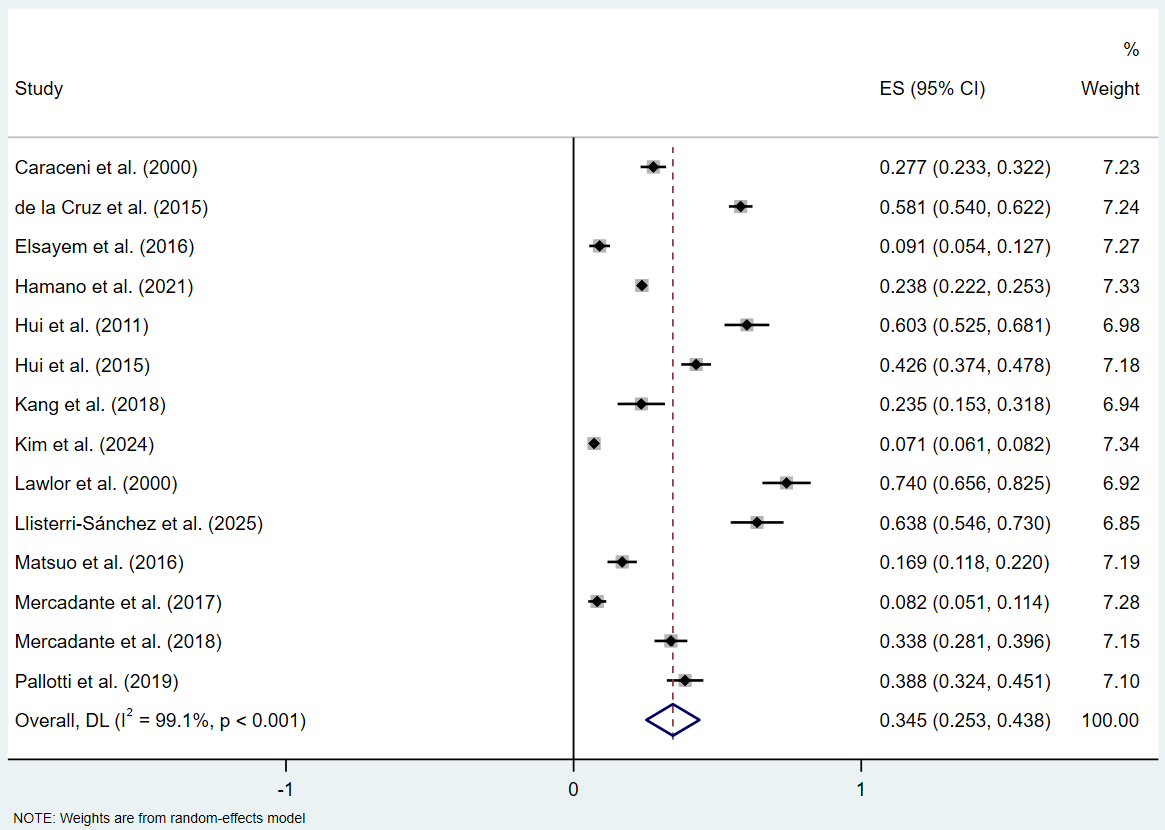


Figure S9. Forest plot of the pooled prevalence of delirium after excluding studies in developing countries.


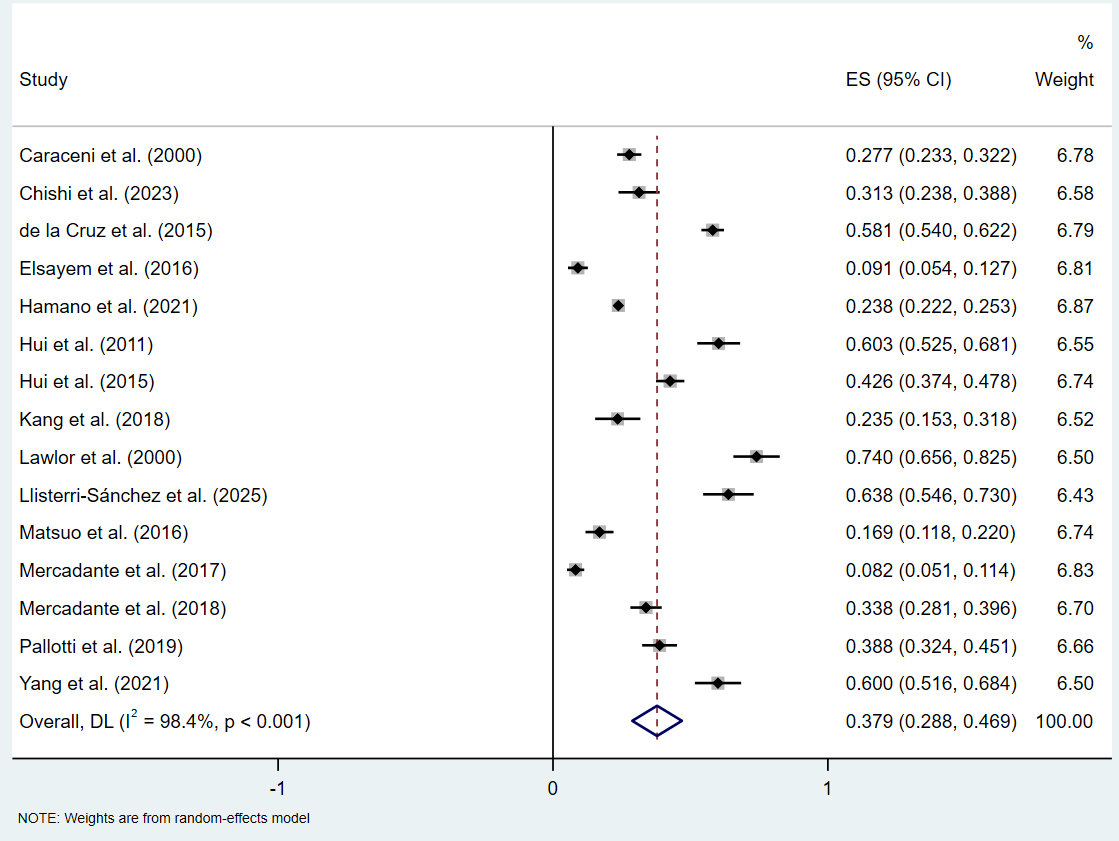


Figure S10. Forest plot of the pooled prevalence of delirium after excluding studies without validated tool.


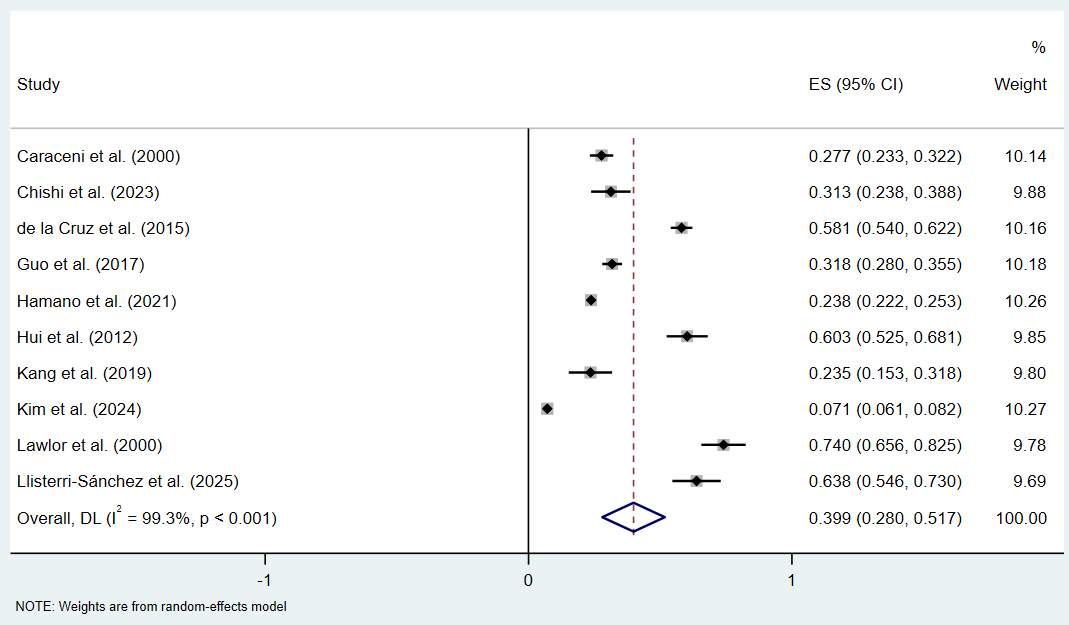


Figure S11. Forest plot of the pooled prevalence of delirium after excluding studies without clear diagnostic criteria.
